# The Impact of Compassion from Others on Organisational Mental Health and Corporate Relations

**DOI:** 10.1192/j.eurpsy.2026.10465

**Published:** 2026-07-31

**Authors:** C. Lazzari, P. Crawford, Y. Kotera

**Affiliations:** 1 Psychiatry, International Centre for Healthcare and Medical Education (ICHME), London; 2 Health Sciences, University of Nottingham, Nottingham, United Kingdom; 3 Center for Infectious Disease Education and Research, University of Osaka, Osaka, Japan; 4 Department of Social Sciences, Azerbaijan University, Azerbaijan, Azerbaijan

## Abstract

**Introduction:**

This study explores how working-age adults view compassion within organizational relationships, focusing on its importance and the belief systems that shape its interpretation. Although research on compassion has grown, understanding of how empathic behaviors are perceived and expected in workplace settings remains limited.

**Objectives:**

This study aims to examine how working-age adults perceive the importance of compassion within organizational relationships and assess the influence of religious and secular belief systems on these perceptions. By analyzing how compassion is recognized, applied, and valued in workplace interactions—especially during moments of crisis—the research seeks to identify the behavioral foundations that support team cohesion and the overall wellbeing of professional environments.

**Methods:**

To explore this, a sample of 274 U.S. adults aged 18 to 65—representing all genders—was recruited through opportunistic, anonymous online sampling to investigate perceptions of compassion in corporate environments. Statistical analysis used the Chi-square Goodness of Fit Index (GFI) to assess response distribution, while Cohen’s *w* measured the effect size, with w values above 1.0 indicating a high impact.

**Results:**

Most participants (59.85%) rated compassion in team relationships as “very important,” and 32.12% as “important,” with no one viewing it as unimportant (GFI: p < 0.001; w > 3.0). During crises, 41.24% felt consistently treated with compassion, 23.72% often, and 26.28% sometimes, with minimal reports of absence (GFI: p < 0.001; w = 2.01). As a tool for team building, 89.05% agreed on its relevance, with few dissenting (GFI: p < 0.001; w > 3.0). Commonly associated behaviours included active listening (80.66%), empathy (65.69%), and supportive communication (62.41%) (GFI: p < 0.001; w = 1.34). Additionally, 91.97% agreed that compassion enhances team performance (GFI: p < 0.001; w > 3.0).

**Conclusions:**

These findings emphasize the vital role of compassion in shaping team dynamics and organizational well-being. Compassionate behaviour—particularly during periods of crisis—not only improves interpersonal relations but also strengthens collaboration and performance. Participants’ emphasis on active listening, empathy, and supportive communication highlights the behavioural foundations of compassion in the workplace. Overall, the study affirms the central role of compassion in fostering relational trust and resilience within corporate structures.

**Disclosure of Interest:**

None Declared

